# Supplementary material for: Effects of Soil Nutrients on Plant Nutrient Traits in Natural Pinus tabuliformis Forests
Source: Plants (Basel). 2023 Feb 7;12(4):735. doi: 10.3390/plants12040735 (PMC9967982; doi:10.3390/plants12040735)
Supplement: Supplementary file 1 [file plants-12-00735-s001.zip › plants-2173315-supplementary.pdf]

plants

MDPI

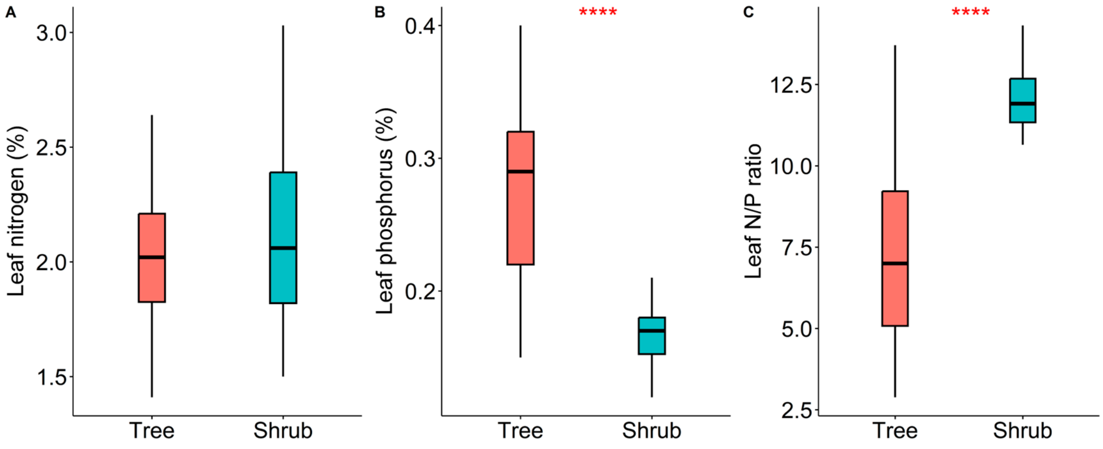

Figure S1. A comparison of the differences in the N_{mass} (A), P_{mass} (B), and N/P (C) at different life forms (Tree and Shrub), **** represents $p < 0.0001$.
